# Interaction of the Koinobiont Parasitoid *Microplitis rufiventris* of the Cotton Leafworm, *Spodoptera littoralis*, with Two Entomopathogenic Rhabditids, *Heterorhabditis bacteriophora* and *Steinernema carpocapsae*

**DOI:** 10.1673/031.013.8401

**Published:** 2013-09-12

**Authors:** Atwa A. Atwa, Esmat M. Hegazi, Wedad E. Khafagi, Gehan M. Abd El-Aziz

**Affiliations:** 1 Deanship of Scientific Research, King Abdulaziz University, Jeddah, Kingdom of Saudi Arabia; 2 Plant Protection Research Institute, Dokki, Giza, Egypt; 3 Department of Economic Entomology, Faculty of Agriculture, Alexandria University, Alexandria, Egypt; 4 Plant Protection Research Institute, Alexandria, Egypt

**Keywords:** competition, entomopathogenic nematodes, intraguild predation, reproduction

## Abstract

Entomopathogenic nematodes are generally considered beneficial organisms. However, they can affect beneficial insects such as parasitoids. The interaction between the entomopathogenic nematodes *Heterorhabditis bacteriophora* Poinar (Rhabditida: Heterorhabditidae) and *Steinernema carpocapsae* Weiser, and the parasitoid *Microplitis rufiventris* Kokujev (Hymenoptera: Braconidae) was investigated in the laboratory. In non-parasitized hosts, *Spodoptera littoralis* Boisduval (Lepidoptera: Noctuidae) larvae exposed to *H. bacteriophora* showed a higher percent mortality than those exposed to *S. carpocapsae*. Both nematodes were able to invade and propagate in non-parasitized *S. littoralis* larvae and those parasitized *by M. rufiventris*. Both nematode species reproduced in *Microplitis*-parasitized hosts, but there was a higher number of nematodes in non-parasitized larvae. *S. carpocapsae* yielded higher numbers of infective juveniles than *H. bacteriophora*. Generally, the number of nematodes harvested increased as their host's size increased. The interaction between the nematodes and parasitoid favored the nematodes when the nematodes were inoculated during the parasitoid egg stage or the young parasitoid larvae, thus giving the nematodes a better chance to grow and reproduce, resulting in the death of the parasitoid larvae. Conversely, when the nematodes were inoculated during the late larval instar of the parasitoid, the competition partially favored the wasp, thus giving approximately 50% of the wasps a better chance to develop, emerge, and reproduce, providing evidence that both nematodes and wasps could reproduce in the same host. Egg maturation of female wasps derived from nematode-infected hosts was not significantly different than those from control hosts. The combined application of nematodes and parasitoids may be beneficial if the detrimental effects of the nematodes on the parasitoid could be avoided by precisely timing the application strategies. It is clear that *Microplitis* larvae and the nematodes share the host larva and engage in a trophic interaction with each other. Intraguild predation is briefly discussed.

## Introduction

The cotton leafworm, *Spodoptera littoralis* Boisduval (Lepidoptera: Noctuidae), is a major plant pest that causes substantial economic losses worldwide. Most control strategies involve chemical insecticides, but this approach is becoming less attractive (Wang et al. 1995) due to resistance, cost, and the lack of availability of pesticides (Mosallanejad and Smagghe 2009). Therefore, biological control has the potential to be a useful strategy. *Microplitis rufiventris* Kokujev (Hymenoptera: Braconidae) is a dominant parasitoid of *S. littoralis, S. exigue*, and *Helicoverpa zea* in Egypt (Hammad et al. 1965). This wasp is a specialist endoparasitoid of earlier instars of *S. littoralis* (late 1^st^ to 3^rd^ instar larvae), when they still live in clusters near the place of egg deposition. However, 3^rd^ instars are preferred. Later instars (4^th^ through 6^th^) disperse, hide under the soil surface in the daytime, and are active at night. As a result, 4^th^ instars are less suitable and less easily accessible than earlier instars for *M. rufiventris* larval development (Hegazi et al. 1977). There is no literature to suggest that *M. rufiventris* females normally attack 5^th^ or 6^th^ instar hosts in the field. The non-preference of later instars has been explained by physiological and host defense traits (Hegazi and Khafagi 2005). The parasitoid oviposits a single egg per host and hasthree instars that feed on the host hemolymph (Hegazi and Führer 1985).

Among the alternative measures to chemical control of insect pests, in recent years attention has focused on biological control using entomopathogenic nematodes of the families Steinernematidae and Heterorhabditidae (Gaugler 1981; Kaya 1985; Poinar 1986 Georgis et al. 2006). These nematodes have a mutualistic symbiosis with a bacteria (*Xenorhabdus* spp. and *Photorhabdus* spp. for *steinernematids* and *heterorhabditids*, respectively) (Poinar 1990). The third stage, infective juveniles, of the nematodes carries the symbiotic bacteria (*Xenorhabdus* in *Steinernemd*) in a special intestinal vesicle (Poinar 1979; Akhurst 1983; Bird and Akhurst 1983), whereas *Photorhabdus* are primarily located in the anterior part of the guts of the *Heterorhabditis indica* infective juvenile (Boemare et al. 1996). The infective juvenile nematodes are attracted to the insects (Gaugler et al. 1980) and enter via the mouth, anus, or spiracles (Mrácek et al. 1988). *Heterorhabditis* infective juveniles are also able to enter through the insect's cuticle (Bedding and Molyneux 1982). They penetrate the hemocoel and release the symbiotic bacteria into the insect's hemolymph. The bacteria then multiply and kill the insect host within 24 hr. Products based on *Steinernema* (*=Neoaplectana*) *carpocapsae* Weiser (Rhabditida: Steinernematidae), *S. feltiae* (*=bibionis*) Bovien (Rhabditida: Steinernematidae) and *Heterorhabditis bacteriophora* (=*heliothidis*) Poinar (Heterorhabditidae) are the most widely commercialized and have almost entirely been marketed as inundative applications in high value niche and specially markets (Ehlers 1996; Georgis et al. 2006). The pests commonly controlled include soil or root-dwelling pests, and the use of nematodes against above ground pests remains negligible, despite a demand for effective microbial sprays against foliar pests (Cross et al. 1999; Copping and Menn 2000). *S. littoralis* has been shown to be susceptible to nematode infection (Sikora et al. 1979), so we therefore selected *S. littoralis* and its braconid, *M. rufiventris*, as a model system to study the interaction between entomopathogenic nematodes and this koinobiont parasitoid.

## Materials and Methods

### Insects

**Rearing of *S. littoralis* and *M. rufiventris*.** Cultures of *S. littoralis* and the parasitoid *M. rufiventris* were obtained from a laboratory colony established in 2009 at the Department of Entomology, Faculty of Agriculture, Alexandria University. The colony of *S. littoralis* and *M. rufiventris* originated from fieldcollected individuals from crops that included cotton in Alexandria, Egypt. However, feral individuals were added to the colonies twice a year to maintain genetic diversity. Larvae of *S. littoralis* were reared on an artificial diet (Hegazi et al. 1977) at 27 ± 1°C, 60–65% RH, and a 14:10 L:D photoperiod. The *M. rufiventris* colony was maintained using 3^rd^ instar *S. littoralis* larvae as hosts, according to methods described by Hegazi and ElMinshawy (1979). Development of *M. rufiventris* from egg to larval maturity is 8–9 days at 27° C and 65 ± 5% RH (Hegazi and Führer 1985). Under these conditions, the parasitoid egg hatches within a day to a mandibulate 1^st^ instar, which roams inside the host for approximately 4 days, eliminating competitors before developing to a 2^nd^ instar. The 2^nd^ instar molts within 12–16 hr into a final 3^rd^ instar, which lasts for 3 days. The last instar exits the host larva and pupates in a silken cocoon near the host. The host larva does not feed or develop further and dies within 3–12 days (Hegazi and Fuhrer 1985). Mating in *M. rufiventris* wasps occurs as soon as both sexes are present (Hegazi et al. 1977), thus male and female wasps grouped together in glass vials (25 × 100 mm) for 24 hr were presumed to have mated. Groups of presumed mated females (hereafter referred to as mated females) were maintained together with the accompanying males throughout the test period. The wasps were provided with fine droplets of honey diluted (1:1) with distilled water daily to ensure maximum reproductive success.

### Nematode cultures

The greater wax moth, *Galleria mellonella* (L) (Lepidoptera: Pyralidae), used as a host for nematodes, was obtained from infested hives and reared on an artificial diet at a constant temperature of 27 ± 2° C and 65 ± 5 % RH, as described by Singh (1994). The final instar larvae (25 days old) were utilized for mass rearing of entomopathogenic nematodes. The entomopathogenic nematodes *H. bacteriophora* (Isolate EBN 10K) and *S. carpocapsae* (Isolate EGB5) were isolated from soil samples in El-Nubaria, Behera, and El Badrashin, Giza, Egypt, respectively (Atwa 1999). The nematodes were cultured in the last instar larvae of *G. mellonella*, according to the methods reported by Kay a and Stock (1997). The infective juveniles of both nematodes were harvested in nematode White traps as described by White (1927) at 25 ± 1° C.

A stock suspension of infective juveniles in distilled water was stored at 10° C for 2 weeks before use. Plastic Petri dishes (9 cm Χ 1.5 cm) lined with filter paper were inoculated with 1200 infective juveniles in 1 mL of water per dish and given 30 minutes to distribute on the filter paper. Each dish was provided with fresh diet (1 Χ 1 Χ 2 cm). Five newly molted 3^rd^ instar *S. littoralis* larvae were added to one dish inoculated with *H. bacteriophora* nematodes, and a second set of five *S. littoralis* larvae was added to the second dish with *S. carpocapsae* nematodes. As a control, the mortality of *S. littoralis* larvae was followed on filter paper inoculated with 1 mL of distilled water without nematodes in a Petri dish. Ten replicates were used for each treatment.

The dishes were maintained in a climate control chamber at 25° C and allowed to incubate for 24 hr, after which the larvae were transferred to rearing cups with a fresh diet. Dead *S. littoralis* larvae, 2–3 days post-treatment, from each replicate were transferred to White traps, and the number of infective juveniles produced was counted (Woodring and Kaya 1988). The infective juveniles were collected daily for one week (Shamseldean et al. 1999). The total number of infective juveniles per White trap was divided by the number of nematode-infected *S. littoralis* larvae to obtain the yield per larva.

### Bioassay methodology

A series of experiments was conducted to determine how *M. rufiventris* might interact with the *H. bacteriophora* or *S. carpocapsae* nematodes when two species that are competing for the same prey attack the same *S. littoralis* larva. Groups of 30 3^rd^ instar *S. littoralis* larvae (determined by the presence of a molted head capsule) were prepared. These larvae were presented individually to female wasps. Ovi-position by females (1–2 days old) was observed for individual female in 15 Χ 60 mm Petri dishes (5–7 females/dish), and only one oviposition was allowed per host larva. Nematode treatments started on days 0, 3, 5, or 7 after parasitism (i.e., times to coincide with the occurrence of egg stage, mid 1^st^, 3^rd^, and late 3^rd^ instar of the parasitoid in the host, respectively). The weights for each parasitized *S. littoralis* host in each test category and those of age-matched non-parasitized hosts were performed to test the hypothesis that infective juvenile production is related to the initial weight of the hosts upon nematode infection.

After each treatment, 10% of each treated group was dissected to ascertain the parasitoid's developmental stage. If 70% of dissected insects showed the same immature stage, nematode treatment of the corresponding test group would be designated for that stage of development. The larvae were allowed to feed on diet *ad libitum* for 24 hr, after which time the treated parasitized larvae were reared on a nematode-free diet under the environmental conditions mentioned above until the host died, pupated, or the parasitoid emerged. Mortality counts were recorded daily for five to seven days from the initiation of the experiment. The nematode yield per larva was recorded as mentioned above. As a control, we also determined the mortality of nonparasitized larvae on filter paper inoculated with 1 mL distilled water without nematodes in a Petri dish. Newly formed parasitoid cocoons from nematode-treated host larvae were collected and checked daily for adult emergence. The externally unaffected (normal) parasitoid females obtained from the treated hosts were collected, grouped in pairs, and placed in glass vials (10.3 by 2.3 cm). This was achieved by pairing a female, which resulted from a treated and surviving host larva, with two males grown using normal laboratory cultures. Honey droplets were smeared on the inner surface of the lid of the rearing vials. Ten females (1 day old) from each nematodetreated group were removed and submersed in 70% ethanol for 10 minutes. Their reproductive tracts were dissected in saline solution. The ovaries were dissected under a binocular dissecting microscope at 40Χ into the egg tube, reservoir, and calyx. Similarly sized developing eggs were gently teased separately from the egg reservoir and calyx. To standardize the egg counting, only eggs that possessed a distinct opaque area (380–390 µm in length) were counted.

### Statistics

The experimental design was completely randomized and balanced (equal numbers of subjects were assigned randomly to each treatment group). The data presented as percentages were normalized using a logarithmic transformation. Data were subjected to analysis of variance (one-way ANOVA) for determination of differences between means. Where significant differences occurred, a least significant differences test was applied for mean separation. The level for significance testing was set *at p* < 0.05 (Winer et al. 1991). Duncan's multiple range test or Student's ttest were applied to significant differences for mean separation. Parameter estimates are given as mean ± 1 SEM unless otherwise stated. (Steel and Torrie 1986).

**Table 1.**
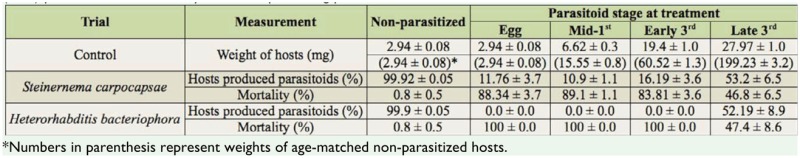
Table 1. Effects of the timing of the nematode application against *Spodoptera littoralis* parasitized larvae on the percent (± SE) parasitized host mortality and hosts producing parasitoids.

## Results

### Effect of nematodes on the mortality of *S. littoralis* larvae

**Figure 1.**
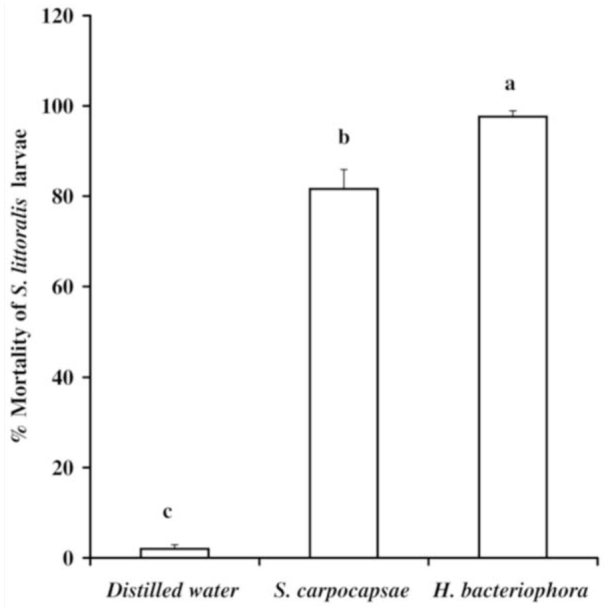
Mean (± SE) percent mortality of 3^rd^ instar *Spodoptera littoralis* larvae exposed to *Steinernema carpocapsae* and *Heterorhabditis bacteriophora* nematodes. Bars bearing the same letter are not significantly different by ANOVA (*p* < 0.01). High quality figures are available online.

Mortality of non-parasitized *S. littoralis* larvae after exposure to nematodes occurred only between 24 and 72 hr. & *littoralis* larvae infected with *S. carpocapsae* retained their color, whereas those infected with *H. bacteriophora* developed a red-brown color, which is characteristic of *Heterorhabditis*-*infected G. mellonella* (Woodring and Kaya 1988). The rate of larval mortality (Figure 1) in the *H. bacteriophora* treatments was significantly higher (97.6%) than in the *S. carpocapsae* treatments (81.6%) (F = 23.68, df = *2.27, p* < 0.01). The mortality and production of parasitoid progeny for the parasitized *S. littoralis* larvae infected by nematodes at 0, 3, 5, or 7 days post-parasitism are shown in Table 1. Host mortality occurred between 24 and 120 hr. Analysis of variance showed that the percent mortality resulting from the treatments was significantly different (for *Steinernema*, F = 111.202, df = 4.45, *P >* 0.01; for *H. bacteriophora*, F = 186.89, df = 4.45, *p* < 0.01). When the nematode *S. carpocapsae* was applied on parasitized hosts either on day 0 or 3 post-parasitism, the percent mortality (Table 1) was slightly increased compared to nonparasitized hosts (Figure 1). However, a significant reduction in the larval mortalities was detected when the nematode treatments were applied on day 7 post-parasitism (Table 1). *H. bacteriophora* nematode treatments on *S. littoralis* larvae on day 0, 3, or 5 post-parasitism resulted in 100% mortality. In contrast, when nematodes were applied on hosts containing late 3^rd^ instars of parasitoid larvae (day 7 postparasitism), host mortality was significantly reduced by more than 53%. The number of nematode-treated hosts that produced wasps was used to determine the sensitivity of *M. rufiventris* eggs or wasp larvae to nematode infection (Table 1). The mean number of wasps that completed their development under various post-parasitism treatments was significantly different (for *S. carpocapsae*, F = 124.729, df = 4.45, *p* < 0.01; for *H. bacteriophora*, F = 203.793, df = 4.45, *p* < 0.01). Exposure of developing wasps in the egg stage, mid 1^st^, and early 3^rd^ larval stages to *S. carpocapsae* infective juveniles via their hosts resulted in a low percentage of hosts that produced wasps (11.7, 10.9, and 16.19%, respectively). However, full-grown parasitoid larvae were partially protected from nematode infection, as 6.5 ± 2.53% completed their development and successfully emerged from treated hosts. When the *H. bacteriophora* infective juveniles were applied on *S. littoralis* hosts either on day 1, 3, or 5 post-parasitism, none of the hosts produced parasitoids. In contrast, when *H. bacteriophora* infective juveniles were applied to hosts containing older parasitoid larvae (late 3^rd^ instars), 52.19% of parasitized hosts produced parasitoids. Some of the wasp larvae that emerged from nematode-parasitized *Spodoptera* larvae (9.6 ± 2.6%) did not form cocoons and died within 24–48 hr of emergence.

**Figure 2.**
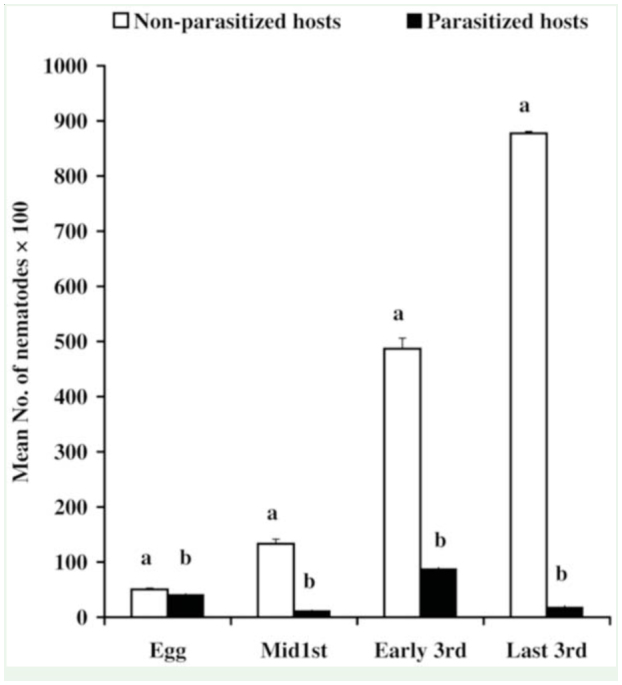
Mean number (± SE) of *Steinernema carpocapsae* nematode yields in non-parasitized and parasitized *Spodoptera littoralis* larvae by *Microplitis rufiventra* wasps. For each set, bars bearing the same letter are not significantly different at *p* < 0.01. High quality figures are available online.

**Figure 3.**
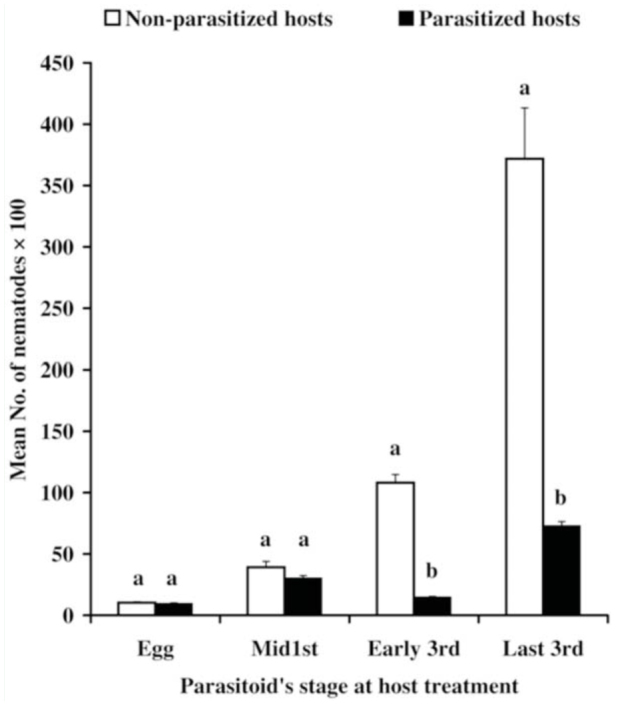
Mean number (± SE) of *Heterorhabditis bacteriophora* nematode yields in non-parasitized and parasitized *Spodoptera littoralis* larvae by *Microplitis rufiventrise* wasps. For each set, bars bearing the same letter are not significantly different at *p* < 0.01. High quality figures are available online.

### Nematode yields in parasitized *S. littoralis* larvae

The yield of nematode infective juveniles in parasitized and non-parasitized *S. littoralis* larvae is shown in Figures 2 and 3. Significant differences were detected in *S. carpocapsae* nematode among parasitized hosts (F = 284.409, df = 3.36, *p* < 0.01). Application of infective juveniles against host larvae on days 0, 3, 5 or 7 post-parasitism produced (± SE) 4094 ± 163, 1169 ± 135, 8790 ± 231, and 1825± 261 infective juveniles per larva, respectively. Corresponding age-matched nonparasitized hosts produced significantly higher numbers of nematodes, with 5045 ± 249, 13,328 ± 821, 48,388 ± 1928, and 87,747 ± 3456 infective juveniles per larva, respectively. In all the *S. carpocapsae* treatments, the number of nematodes harvested was significantly higher in non-parasitized larvae compared to parasitized larvae (t_0.05_ = 3.3, df = 18, t_0.05_ = 14.607, df = 18, t_0.05_ = 60.708, df = 18 and t_0.05_ = 4.787, df = 18, for hosts treated at days 0, 3, 5, or 7 post-parasitism, respectively). *H. bacteriophora* reproduced in the & *littoralis* host larvae but at a lower multiplicative rate than *S. carpocapsae*. Significant differences were recorded in nematode yields between the different postparasitism ages of parasitized host larvae (F = 193.985, df = 3.36, *p* < 0.01). When *H. bacteriophora* infective juveniles were applied on day 0, 3, 5, or 7 post-parasitism, the larvae produced 951 ± 55, 3033 ± 205, 1471 ± 74and 7289 ± 345 infective juveniles, respectively, versus 1033 ± 66.9, 3913 ± 483, 10,814 ± 673, and 37,184 ± 4152 infective juveniles per age-matched non-parasitized host, respectively. When comparing the nematode yields between parasitized and non-parasitized *S. littoralis* larvae, there were no significant differences in nematode propagation when the nematodes were applied on hosts either 0 or 3 days post-parasitism. However, significant differences were detected when the nematodes were applied on day 5 (t_0.05_ = 17.79, df = 18) or day 7 post-parasitism (t_0.05_ = 7.18, df = 18).

### Egg maturation of female wasps derived from nematode-infected hosts

**Figure 4.**
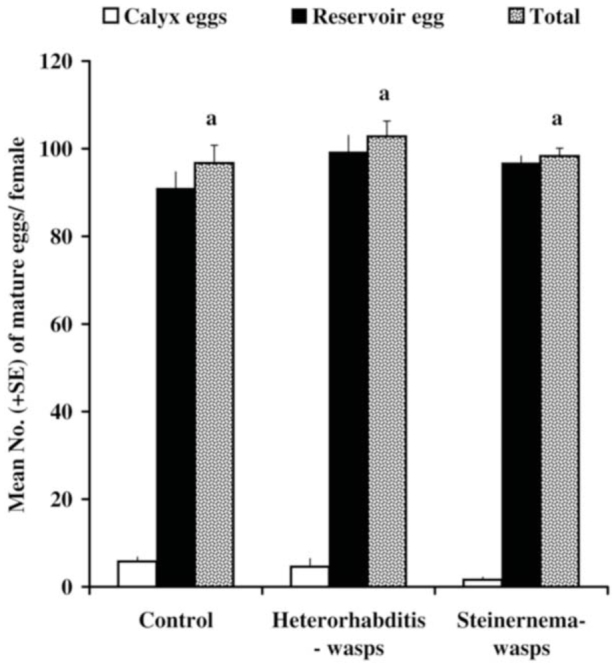
Mean number (± SE) of mature eggs and their relative distribution in calyx lumen and oviducts of 1-dayold *Microplitis rufiventris* females derived from *Spodoptera littoralis* larvae treated with *Heterorhabditis bacteriophora* and *Steinernema carpocapsae* nematodes. High quality figures are available online.

Adult *M. rufiventris* females developing from nematode treated *S. littoralis* larvae on days 5 or 7 post-parasitism that appeared morphologically normal were able to find and attack their hosts. The dissected ovaries of mated, but host-deprived, parasitoid females (1 day old) that resulted from nematode treated hosts did not show a significantly different number of mature oocytes (Figure 4) compared to control females. The dissection of some mature, full-grown parasitoid larvae derived from nematode-treated hosts containing early 3^rd^ or late 3^rd^ instar parasitoid larvae upon treatment showed that they were nematode free. The oviducts of the adult wasps derived from the control, *Heterorhabditis-* and *Steinernema*treated hosts, contained a non-significant number of mature eggs (96.7 ± 4.09, 102.8 ± 3.5, and 98.3 ± 1.8 eggs/female, respectively).

## Discussion

When *S. carpocapsae* and *H. bacteriophora* were screened for their efficacy against 3^rd^ instar larvae of *S. littoralis* using a filter paper bioassay, both nematode species were able to invade and propagate in the larvae tested. The obtained results indicate that *H. bacteriophora* induced significantly higher percent mortality than & *carpocapsae*. The results are consistent with previous results reported by Abdel-Kawy et al. (1992), who stated that the 3^rd^ and 4th instar of *S. littoralis* larvae were susceptible to infection even at the lowest inoculum levels. Sikora et al. (1979) showed that most developing stages of *S. littoralis* were susceptible to *S. carpocapsae* infection, with the exception of the pre-pupal stage. However, neonate larvae of *S. exigua* were also significantly susceptible to nematode infection of *S. feltiae* (Kaya 1985).

The number of nematode generations inside the host can change according to the different hosts, the size of the host, food availability, the number of infective juveniles that penetrated the host, and the environmental conditions (Griffin et al. 2005; Bazman et al. 2008). The multiplication of nematodes against & *littoralis* larvae was examined by comparing the number of nematodes produced between parasitized and non-parasitized larvae. The obtained results suggest that the number of nematodes harvested was directly proportional to the weight of the larvae.

The nematode yields were higher in nonparasitized *S. littoralis* larvae than in parasitized larvae. The parasitoid *M. rufiventris* is a polyDNA virus carrying braconid wasp. *S. littoralis* larvae parasitized by this wasp exhibit reduced growth (Hegazi et al. 2005). Therefore, the higher yields of infective juveniles in control hosts may be attributed to larger body weights of non-parasitized larvae compared to parasitized larvae. In all cases, *S. carpocapsae*-infected larvae yielded a higher number of nematodes than *H. bacteriophora*-infected larvae.

The results suggest that in *S. littoralis* larvae, *S. carpocapsae* reproduced more than *H. bacteriophora*. When the eggs and young larvae of *M. rufiventris* were exposed to *S. carpocapsae* or *H. bacteriophora* via *S. littoralis* larvae, the combined application resulted in a higher percent mortality of host larvae compared to the use of either the parasitoid or nematode alone. A higher host-insect mortality was previously observed when entomopathogenic nematodes were combined with parasitoids. For example, Mbata and Shapiro-Ilan (2010) reported that a combination of the nematode *H. indica* and the parasitoid *Habrobracon hebetor* increased the mortality of *Polidia interpunctella*. Additionally, Dillon et al. (2008) observed that the interaction between the nematodes *H. downesi* or *S. carpocapsae* and the parasitoid *B. hylobii* enhanced the mortality of the host insect, *Hylobius abietis*.

Entomopathogenic nematodes are known to have an adverse effect on the development of some parasitoids (e.g., Head et al. 2003; Lacey et al. 2003). When the nematodes *Heterohabditis downesi* were applied to the gregarious ectoparasitoid *B. hylobii*, which feeds on larvae of the weevil *Hylobius abietis*, the nematodes parasitized the parasitoid larvae, and there was a reduction in parasitoid cocoon formation and fewer cocoons that were enclosed (Everard et al. 2009). It is clear from the experiments reported here that *S. carpocapsae* and *H. bacteriophora* have an impact on the internal developmental stages of *M. rufiventris*. Parasitoid eggs were adversely affected by the nematode treatments, which were initiated concomitant with parasitization. Additionally, if the wasps were in the 1^st^ to early 3^rd^ larval instar stage when *S. littoralis* larvae were parasitized by nematodes, all the wasp larvae in larvae inoculated with *H. bacteriophora* and most inoculated with *S. carpocapsae* died from starvation. The results suggest that nematodes and their associated bacteria rapidly occupy the host larva, dramatically altering its quality for the other organism (*M. rufiventris*). This alternation in resources would effectively starve the young wasp larvae that are not directly killed by nematodes (Everard et al. 2009).

The secondary metabolites produced by entomopathogenic nematodes might also act as antagonistic factors that hinder the development of the young wasps. Therefore, the nematode exclusively developed in the host and induced high mortalities. Parasitoid death due to premature death of the host is the most common consequence of a host-parasitoidpathogen interaction and has been reported in several laboratory studies using nematodes, primarily *S. carpocapsae*. This is particularly clear in cases where the parasitoid itself is not infected by the nematodes, which was the case for the wasp in our study and the endoparasitoid braconids *Glyptapanteles miliraris* (Kaya 1978), *Apanteles ultor* (Triggiani 1985), and *Myxexoristops* sp. (Mrácek and Spitzer 1983). In our study, the premature death of the host is the most likely cause of parasitoid failure in the experiments where the nematodes were applied on day 0, 3, or 5 post-parasitism. The effect of nematodes on the young stages of *M. rufiventris* appears to be directly antagonistic, the costs of which may be measured in terms of the loss of progeny that fail to complete development, reduced adult size, and increased development time (data not shown). However, when the nematode treatments were performed on the *S. littoralis* larvae on day 7 post-parasitism, the nematodes were not effective in preventing all the parasitoid larvae from emerging, but the emergence time was delayed by two days and costs were less. The full-grown parasitoid larvae were almost completely protected from nematode penetration within their hosts. The reduced sensitivity of late stage parasitoid larvae to nematode infection may make the two compatible in an integrated control program for *S. littoralis* because the nematodes do not kill all the parasitoids.

These findings suggest that the various developmental stages of the parasitoids have varying susceptibilities to entomopathogenic nematodes, thus confirming an earlier observation that later parasitoid stages are less affected by entomopathogenic nematodes than earlier parasitoid stages (Kaya 1987). The later parasitoid stages may have already developed an effective immunity strategy against nematode infection. The effects of nematode infection were more evident in parasitoid adults that were exposed to nematodes while early 3^rd^ instar. The survival of these adults was significantly reduced (*p* < 0.05) (data not shown). 8–10% percent of the apparently normal resultant adults died within a few days after emergence. When host larvae were introduced to these adults, no parasitiza-tion occurred. However, nematode-treatments against late 3^rd^ instar wasp larvae were not effective in preventing significant numbers of wasp larvae from completing their development and emerging.

The *S. littoralis* larvae develop through six larval instars. The first three larval instars feed in groups, leaving the opposite epidermis of the leaf intact. *M. rufiventris* can attack these hosts. The 4^th^ to 6^th^ instar larvae disperse and spend the day in the ground under the host plant, where entomopathogenic nematodes may exist, feeding on plant leaves at night and early in the morning. There is little information on the response of parasitoids to nematode-infected hosts. In the present work, both parasitoid and nematodes targeted the *Spodoptera* larvae, and so there is potential for competition or intraguild predation when two species competing for the same prey attack and consume the food of the one another. Intraguild predation occurs when two species that share a host also engage in a trophic interaction (predation or parasitism) with each other (Rosenheim et al. 1995). Specifically, the entomopathogenic nematodes *H. bacteriophora* or *S. carpocapsae* infect the parasitized host larvae did not infect the mature parasitic larvae, and the presence of nematodes along with the younger parasitic larvae decreased the chance of wasp survival to adulthood. Nonetheless, using both the parasitoid and entomopathogenic nematodes together results in greater overall mortality on *S. littoralis* larvae than either agent inflicts alone. In entomopathogenic nematodeparasitized hosts there was a significant reduction in wasp's cocoon formation, or no cocoons eclosed at all. Entomopathogenic nematodes are known to interact antagonistically with other competitors, such as entomopathogenic fungi (Barbercheck and Kaya 1991) and parasitoids (Sher et al. 2000;Stuart et al. 2006). The ichneumonids *Mastrus ridibundus* and *Liotryphon caudatus* avoided codling moth hosts previously exposed to *S. carpocapsae* nematodes (Lacey et al. 2003).

No significant differences were observed between the number of mature eggs in the oviducts of 1-day-old females derived from treated hosts and those from non-treated hosts. This study provides evidence that both nematodes and wasps can reproduce in the same host. Therefore, the costs for the parasitoid associated with nematodes attacking the parasitized hosts are dependent on the timing of the application. The ability of these nematodes to avoid the full-grown wasp larvae and survive nematode treatments in parasitized hosts enhances the complementarity of entomopathogenic nematodes and *M. rufiventris*. The interactions between nematodes and wasps in a single host are relevant to application strategies (Barbercheck and Kaya 1991). We conclude from this study that when nematodes and parasitoids are applied concurrently, both compete for the same host, the costs of which are possibly more severe for the parasitoid.

The dual application of parasitoids and nematodes may result in a more efficient control of insects when they are applied sequentially and with the proper timing. Additional studies are needed to further define the interactions in the parasitoid-nematode community in an agroecosystem.
